# Artificial intelligence in haematology laboratory diagnosis: Current applications, challenges, and future directions

**DOI:** 10.4102/ajlm.v15i1.3130

**Published:** 2026-07-23

**Authors:** Hussam A. Osman

**Affiliations:** 1 Department of Health and Laboratory Science, College of Medical and Health Sciences, Liwa University, Abu Dhabi, United Arab Emirates

**Keywords:** artificial intelligence, machine learning, deep learning, haematology diagnostics, digital morphology, automated cell classification, computational pathology, federated learning

## Abstract

**Background:**

Artificial intelligence (AI) is transforming haematology diagnostics by improving accuracy, efficiency, and reproducibility in workflows traditionally reliant on manual microscopy and expert interpretation. Integrating AI into laboratory medicine presents opportunities to enhance diagnostic precision and reduce variability, particularly in resource-limited settings.

**Aim:**

This narrative review examines the application of AI across major domains of haematology, morphological diagnosis, flow cytometry, cytogenetics, genomics, and clinical decision support, while addressing ethical, regulatory, and economic considerations relevant to global and African laboratory contexts.

**Methods:**

A comprehensive literature search of PubMed, Scopus, Web of Science, and EMBASE identified studies describing or evaluating AI algorithms in haematologic diagnostics, focusing on model performance, validation level, and clinical applicability.

**Results:**

Recent studies demonstrate strong performance of deep learning models, particularly convolutional neural networks and hybrid convolutional neural networks–transformer architectures, in automating blood and bone marrow morphology, detecting subtle dysplastic changes, and supporting digital workflows. In flow cytometry, AI enhances automated gating and rare event detection, while cytogenetic and genomic tools aid in karyotyping, variant classification, and structural abnormality recognition. Decision-support systems further assist in diagnostic triage and treatment planning. However, widespread implementation is limited by data heterogeneity, inadequate multicentre validation, and evolving ethical and regulatory frameworks.

**Conclusion:**

Advancing AI integration in haematology will require robust validation studies, explainable AI approaches, and equitable adoption strategies. Strengthening African laboratory systems through such innovations offers a pathway toward improved diagnostic capacity and sustainable digital transformation.

**What this study adds:**

The current study highlighted that AI is improving haematology laboratory tasks such as cell identification, differential diagnosis assistance, and result interpretation, but still faces challenges in validation, bias, and workflow integration. Future progress depends on stronger external validation, better explainable models, and closer collaboration between laboratory experts and AI specialists.

## Introduction

The diagnosis of haematological disorders traditionally relies on the expertise and visual judgment of pathologists. Conventional laboratory diagnosis, particularly through manual blood smear and bone marrow examination, depends heavily on recognising cellular morphology and pattern variations. While this approach has been central to haematology, it faces persistent challenges such as inter-observer variability, practitioner exhaustion, and inconsistency in complex or borderline cases. Moreover, increasing diagnostic workloads and rising expectations for accuracy continue to expose the limitations of manual microscopy.

Artificial intelligence (AI) offers a promising pathway to address these challenges. Using computer vision, deep learning, and pattern recognition algorithms, AI systems can identify subtle morphological and molecular features that might not be discernible to the human eye. Applications of AI in haematology now extend across digital morphology, flow cytometry, cytogenetics, genomics, and clinical decision support (CDS). These technologies are reshaping haematologic diagnostics by enhancing reproducibility, reducing turnaround times, and complementing expert interpretation. For instance, AI-powered systems currently analyse digital blood smears for cell classification and counting,^1,2^ optimise flow cytometry data for immunophenotyping and detection of minimal residual disease,^3,4^ and assist in genomic variant interpretation and risk prediction.^5,6^

Despite these advancements, transitioning AI from research prototypes to reliable clinical tools remains challenging. Barriers include variability in model development, limited use of multicentre datasets, inconsistent validation, and unclear ethical and regulatory frameworks.^7,8,9,10,11,12,13^ Addressing these gaps is essential for widespread clinical adoption and trust among practitioners.

### African context and relevance

In Africa, the integration of AI into haematology presents significant opportunities to address persistent challenges related to healthcare infrastructure and workforce limitations.^14^ Laboratories in several African countries, including Nigeria, Kenya, and South Africa, often face inadequate infrastructure, shortages of trained personnel, and limited resources. These constraints frequently result in prolonged diagnostic turnaround times and variability in test accuracy and consistency.^15,16^ Despite these challenges, the current landscape provides a strong foundation for AI-driven innovation, offering scalable solutions that can enhance efficiency and accessibility in diagnostic services.^14^

Artificial intelligence-powered digital morphology platforms and image interpretation systems have the potential to decentralise diagnostic capabilities across the continent. By enabling smaller or remote laboratories to access centralised or cloud-based AI platforms, these technologies can improve diagnostic coverage while promoting standardisation of results.^17^ Pilot initiatives in countries such as Kenya and South Africa are already exploring the integration of AI into pathology workflows, with the aim of enhancing diagnostic accessibility and improving consistency in reporting.^17^ Furthermore, the United States Agency for International Development has highlighted AI’s potential to ‘leapfrog’ conventional diagnostic pathways by allowing machine learning (ML) algorithms to screen digitised slides, identify regions of interest for clinician review, or, in some cases, directly support diagnostic decision-making through pattern recognition.^17^

The deployment of AI-based tools for the automated detection of red blood cell–related diseases has also been reported in West Africa.^18^ These systems employ advanced ML techniques to analyse red blood cell morphology and identify abnormalities associated with a range of haematological conditions. Studies integrating artificial neural networks and convolutional neural networks (CNNs) with traditional classifiers such as support vector machines and k-nearest neighbour algorithms have demonstrated enhanced diagnostic performance, with reported accuracies ranging from 91% to 98%. Collectively, these developments underscore the dual role of AI as both a diagnostic support mechanism and a capacity-building tool within African healthcare systems.^16^

This narrative review critically examines current applications of AI in haematology laboratory diagnosis across key thematic domains, morphological diagnostics, flow cytometry, cytogenetics and genomics, and CDS. It also addresses ethical, regulatory, and economic considerations relevant to AI implementation, with particular attention to resource-limited contexts. Finally, the review provides future perspectives for sustainable and equitable integration of AI in global and African haematology practice.

The literature in this field has expanded rapidly in recent years. However, available evidence is scattered across studies with different designs and methodologies. This fragmentation limits clear interpretation and practical application. In addition, findings are sometimes inconsistent and lack unified synthesis. A narrative review is therefore needed to integrate current knowledge, clarify key concepts, and highlight unresolved issues within the field.

This narrative review aims to provide a focused and critical synthesis of the existing literature. It summarises key molecular, diagnostic, and translational findings. It compares results across different study types. It also identifies limitations, knowledge gaps, and priorities for future research.

## Methods

This study adopts a narrative review methodology to synthesise and contextualise current evidence on the application of AI in haematology laboratory diagnostics. The narrative approach was selected due to the heterogeneity of study designs, algorithms, and validation strategies in the existing literature, which limits the feasibility of systematic quantitative synthesis.

Published studies were identified from peer-reviewed literature focusing on AI applications in haematological morphology, flow cytometry, cytogenetics, genomics, and CDS. Both experimental and translational studies were considered. Emphasis was placed on clinically relevant outcomes, algorithmic performance, and implementation challenges. Additional attention was given to studies addressing ethical, regulatory, and economic aspects, as well as reports relevant to African and other resource-limited contexts.

## Results

### Artificial intelligence in morphological diagnosis

The application of AI in morphological haematology has transformed how blood and bone marrow samples are analysed. Artificial intelligence-driven image analysis systems enable objective, high-throughput, and reproducible cellular classification, minimising human bias. Convolutional neural networks have demonstrated remarkable performance in identifying leukocyte subtypes and detecting abnormal morphologies.^19,20,21^ Widely adopted platforms such as CellaVision and Morphogo have achieved classification accuracies of 95% – 98% for standard leukocyte types, matching and sometimes surpassing manual microscopy.^20,21,22,23,24,25,26,27^ These systems not only enhance diagnostic accuracy but also reduce inter-observer variability and optimise technologists’ time by automating routine differential counts.

More advanced hybrid models that integrate CNNs with transformer architectures have further improved recognition of subtle morphological abnormalities. These hybrid systems can detect dysplastic changes characteristic of myelodysplastic syndromes and identify abnormal red cell morphologies such as schistocytes and spherocytes.^28,29,30,31^ Through continuous learning on large annotated datasets, these algorithms capture minute, non-obvious features that may be challenging for humans to interpret consistently, thereby supporting more standardised and reproducible reporting.

Incorporating whole-slide imaging has expanded the utility of AI in digital haematopathology. Modern systems can digitise glass slides into high-resolution images for AI-assisted analysis and pathologist review.^32,33^ This integration enables rapid detection, quantification, and prioritisation of abnormal regions, facilitating both diagnostic accuracy and workflow efficiency. Moreover, cloud-based AI platforms now enable remote consultation and collaborative interpretation, an important advancement for laboratories across Africa, where access to haematopathologists may be limited. By enabling virtual slide review and automated differential analysis, AI-powered whole-slide imaging platforms can bridge geographical gaps and contribute to equitable diagnostic access.

While AI-based morphology systems demonstrate high diagnostic accuracy across multiple studies, most remain confined to controlled research environments. Broader multicentre validation and cost-effectiveness evaluations are still required before widespread clinical deployment, particularly in resource-limited settings.

### Artificial intelligence in flow cytometry

The integration of AI and ML into flow cytometry has revolutionised how complex, high-dimensional data are analysed. Traditionally, manual gating, a subjective, time-consuming process, relied heavily on expert interpretation, often introducing human bias. Artificial intelligence now enables automated, reproducible, and efficient analysis through advanced clustering, visualisation, and classification techniques.

Unsupervised algorithms such as FlowSOM and t-SNE allow for unbiased clustering of cells based solely on marker expression, effectively reducing analyst-to-analyst variability.^34,35^ These tools automate data organisation and visualisation, providing objective identification of cellular subpopulations. Building on this foundation, supervised deep learning models such as DeepFlow learn from expert-defined cell populations and apply this knowledge to new datasets, further standardising results and reducing turnaround time.

Beyond automation, ML has enhanced diagnostic insight: ML models have shown superior performance in detecting rare cell populations and subtle immunophenotypic changes associated with haematological malignancies.^36,37,38^ By integrating multiple parameters simultaneously, these models provide sensitive and quantitative assessments of disease status particularly for detecting minimal residual disease in acute myeloid leukaemia and myelodysplastic syndromes.^36,38^ Artificial intelligence-based minimal residual disease analysis reduces subjectivity and provides robust, reproducible results, which is critical for both initial diagnosis and post-treatment monitoring.

In the African context, AI-enabled flow cytometry offers significant potential to improve access to standardised diagnostic testing. Centralised AI-assisted cytometry platforms could support smaller or under-resourced laboratories by processing uploaded data through secure, cloud-based systems. This approach would allow regional centres, such as those in Nigeria, South Africa, and Kenya, to share computational resources while maintaining local data governance. Such collaborative networks could reduce the current gap in advanced haematology diagnostics across the continent and enhance training capacity for laboratory scientists.

Despite notable success in automating complex flow cytometry data interpretation, algorithmic transparency and clinical validation across diverse populations remain limited. Integration into African laboratory workflows will depend on infrastructure investment and standardised regulatory oversight.

### Artificial intelligence in cytogenetics and genomics

Artificial intelligence applications in cytogenetics and genomics have advanced rapidly, automating complex image interpretation and data analysis tasks once reliant on manual expertise. Deep learning algorithms are now capable of identifying chromosomal abnormalities, predicting pathogenic variants, and integrating multi-omic data to inform diagnosis and prognosis.

In cytogenetics, CNNs have been developed to automate chromosome segmentation and classification. Tools such as KaryoNet and Varifocal-Net can accurately localise metaphase spreads, isolate individual chromosomes, and identify subtle banding pattern abnormalities.^39,40,41,42,43^ These AI-based systems demonstrate accuracy levels exceeding 98% in classifying chromosomal structures, significantly reducing human workload and inter-observer variability. Moreover, they facilitate a human-in-the-loop workflow, allowing cytogeneticists to focus on verifying AI-flagged anomalies, such as deletions, translocations, and duplications.

Artificial intelligence has also expanded into genomic interpretation, particularly for analysing next-generation sequencing data. Models such as AlphaMissense and MissenseNet predict the pathogenicity of missense variants with high accuracy,^44,45,46^ while frameworks like MethylNet and ChINN enable the interpretation of DNA methylation and chromatin interaction data^47,48^ (Table 1). These developments are transforming genomics from descriptive to predictive science, enabling clinicians to infer biological function and clinical impact directly from sequence data.^49,50,51^

**TABLE 1:** Types of AI algorithms in haematology diagnostic platforms, validation level, and implementation status as identified during the current study.

Platform	AI algorithm	Output	Accuracy (%)	Level of validation	Implementation status
Morphology	CNN, ResNet, YOLOX	Cell classification; leukemia subtype recognition	95–98	Multicentre validation studies (Europe, Asia)	Clinically deployed in digital morphology analysers (e.g., CellaVision DM96)
Flowcytometry	DeepFlow, FlowSOM	Automated gating; phenotype discovery	93–96	Multicentre and retrospective validation	Prototype to early-clinical deployment in academic laboratories
Cytogenetics	KaryoNet, Varifocal-Net	Chromosomal abnormality detection	96–98	Single-centre and simulated datasets	Research stage; pilot clinical evaluation
Genomics	MethylNet, ChINN	Variant and methylation analysis	90–94	Multicentre cross-validation on open genomic datasets	Research-to-prototype stage; clinical translation ongoing
Decision support	Ensemble, Reinforcement Learning models	Diagnostic and therapeutic prediction	92–98	Retrospective cohort studies	Prototype: academic research use; not yet clinically certified

Note: Please see the full reference list of Osman HA. Artificial intelligence in haematology laboratory diagnosis: Current applications, challenges, and future directions. Afr J Lab Med. 2026;15(1), a3130. https://doi.org/10.4102/ajlm.v15i1.3130.

AI, artificial intelligence.

**FIGURE 1:**
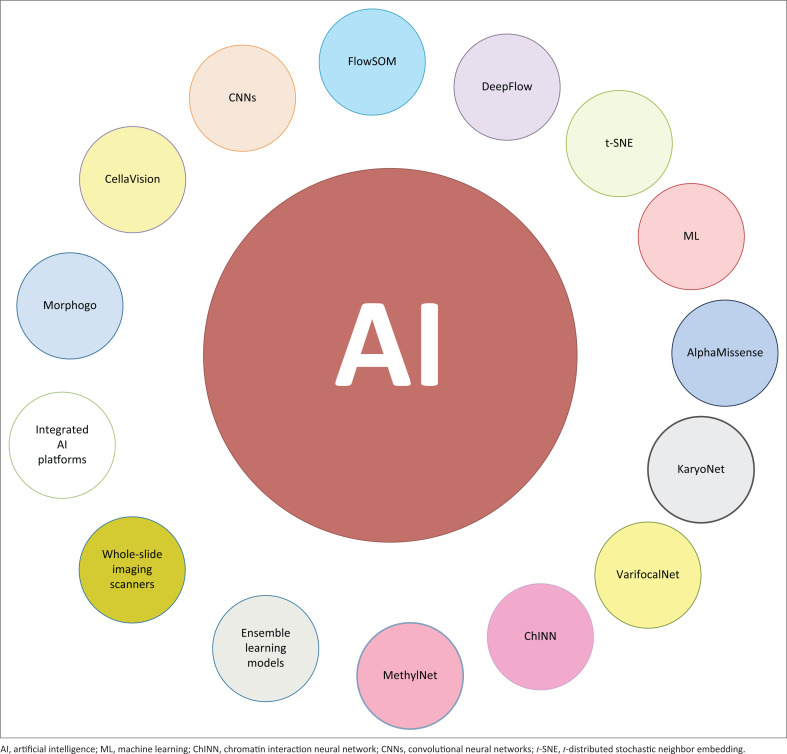
The different types of artificial intelligence and machine learning commonly used in diagnostic haematology as identified during the current study.

In resource-limited settings, including many parts of Africa, the use of AI in cytogenetics and genomics remains limited but promising. Cloud-based AI genomics platforms can offer affordable, scalable tools for laboratories without advanced sequencing infrastructure and preserve data privacy while pooling genomic data across African borders. This strategy could help address the chronic shortage of large, representative datasets required for model validation and ensure that African genomic diversity is adequately reflected in AI-driven analyses (Figure 1).

Current AI-based cytogenetic and genomic tools show strong computational performance but are largely in prototype or academic stages. The translation of these models into clinical diagnostics will require harmonised data governance frameworks and equitable access to genomic resources across low- and middle-income regions.

### Artificial intelligence in clinical decision support

Artificial intelligence is playing an increasingly important role in CDS within haematology. Beyond diagnostic applications, AI models are now being used to assist in therapeutic decision-making, risk stratification, and patient monitoring. By integrating laboratory findings, genomic data, and clinical variables, AI-based CDS systems can enhance personalised treatment approaches and improve workflow efficiency.

Ensemble learning models, for example, have been applied to risk stratification in acute myeloid leukaemia, combining ML outputs with established clinical guidelines to support treatment planning.^52,53^ Reinforcement learning algorithms have further demonstrated potential in optimising dynamic treatment strategies, such as chemotherapy dosing and transfusion management.^54,55,56^ These systems continuously learn from patient data and treatment outcomes, enabling adaptive protocols tailored to individual responses.

Such technologies complement traditional decision-making rather than replace it. By providing evidence-based recommendations and predictive insights, AI-driven CDS platforms allow clinicians to focus on complex cases that require expert interpretation. Importantly, this synergy between clinician expertise and AI analytics enhances both accuracy and efficiency in patient care.

Although decision-support algorithms have demonstrated utility in diagnostic prediction and therapy selection, their ethical governance and cost-effectiveness in routine haematology practice are yet to be established. Regional pilot programmes and transparent validation protocols are essential next steps.

## Discussion

### Ethical, regulatory, and economic factors

The rapid integration of AI into haematology laboratories raises important ethical, regulatory, and health-economic questions that extend beyond technical performance and must be addressed to ensure responsible and equitable implementation. While AI-driven systems demonstrate high accuracy and efficiency, their clinical impact depends on trust, governance, and accessibility within real-world healthcare settings.

#### Ethical and transparency issues

A central ethical concern surrounding AI in haematology is the lack of interpretability associated with many advanced ML and deep learning models. These systems often function as ‘black boxes’, generating outputs without transparent explanations of how decisions are reached.^57,58,59^ Such opacity may undermine clinician confidence; particularly in high stakes diagnostic contexts where accountability and explainability are essential.

Recent advances in explainable AI provide promising pathways to address these concerns. Frameworks such as SHapley Additive exPlanations (SHAP) and Local Interpretable Model-agnostic Explanations (LIME) enable visualisation of model reasoning by identifying the morphological, cytometric, or genomic features that most strongly influence predictions.^57,58,60,61,62,63^ In haematology, the application of explainable AI can enhance interpretability by clarifying how specific cellular features or molecular variants contribute to diagnostic outputs, thereby supporting clinician oversight rather than autonomous decision-making.

Ethical frameworks increasingly emphasise that AI should function as a supportive tool within a human-in-the- loop model. This approach reinforces professional accountability, ensures appropriate clinical judgment, and aligns AI deployment with established ethical principles in laboratory medicine.^64,65,66^ Maintaining human oversight is particularly critical in complex or ambiguous cases where contextual interpretation remains essential.

#### Regulatory landscape

Regulatory oversight of AI-based medical technologies remains heterogeneous across regions, posing challenges for standardised clinical adoption. Regulatory agencies such as the U.S. Food and Drug Administration and the European Medicines Agency have begun developing guidance for software-as-a-medical-device, yet international harmonisation of validation requirements remains limited.^64,65,66^ This fragmentation complicates cross-border deployment and multicentre validation of AI tools.

In Africa, regulatory frameworks for AI in healthcare are still emerging but show increasing momentum. Initiatives supported by the African Union and the World Health Organization African Regional Office aim to establish data governance, ethical safeguards, and algorithmic accountability structures tailored to regional needs.^67^ Collaborative validation studies across African laboratories could play a critical role in developing continent-specific regulatory standards that reflect diverse resource settings, population genetics, and healthcare infrastructures.

#### Health economics and equity

From a health-economic perspective, AI has the potential to reduce long-term diagnostic costs by improving efficiency, standardisation, and workforce utilisation. Automation of routine tasks may reduce reliance on highly specialised personnel and shorten turnaround times. However, initial implementation costs remain substantial, including investments in digital infrastructure, data storage, cybersecurity, and model validation.

Ensuring equitable access to AI technologies will require innovative financing and implementation strategies. Public–private partnerships, regional AI hubs, and shared cloud-based platforms may help distribute costs and expand access, particularly in low- and middle-income countries. Experiences from African countries including Egypt, Benin, Cameroon, Tanzania, Malawi, Kenya, and Nigeria demonstrate how shared digital pathology platforms and centralised AI-supported analysis can reduce costs while improving diagnostic reach.^68^

These examples highlight that sustainable AI integration depends not only on technological capability but also on collaborative implementation models that prioritise scalability, long-term resilience, and equity over short-term efficiency gains. When thoughtfully deployed, AI can strengthen both diagnostic quality and health system robustness, provided that ethical transparency, regulatory rigor, and economic inclusivity guide its adoption.

### Future perspectives

Although AI has already demonstrated substantial promise in haematology diagnostics, several challenges must be addressed before widespread and sustainable clinical adoption can be achieved. Future development should prioritise explainability, generalisability, and ethical accountability to ensure patient safety and clinician acceptance.

#### Advancing technical innovation

Federated learning represents a particularly promising strategy for overcoming data sharing and privacy barriers in AI development. By enabling institutions to collaboratively train models without transferring sensitive patient data, federated learning enhances model generalisability while preserving data sovereignty.^13,69,70,71,72^ This approach is especially relevant in haematology, where data heterogeneity across laboratories can limit reproducibility and external validation.

Within African laboratory networks, federated learning could support the development of inclusive and representative AI models while respecting national data governance regulations. Such collaborative frameworks may help address the chronic underrepresentation of African datasets in global AI research and improve algorithmic fairness.

Explainable AI frameworks will also play a critical role in future implementation. Tools such as SHAP, LIME, and attention-based visualisation methods enhance interpretability and facilitate clinician engagement with AI systems.^60,61,62,63^ The adoption of explainable AI is likely to become a prerequisite for regulatory approval and routine clinical integration, reinforcing trust and accountability.

#### Integrating multi-omics and systems biology

The integration of multi-omics data represents the next frontier in precision haematology. Artificial intelligence-driven pipelines that combine genomic, transcriptomic, proteomic, morphological, and cytometric data offer a systems level understanding of disease biology.^73,74,75,76,77^ This multidimensional approach enables more accurate prediction of disease progression, therapeutic response, and relapse risk.

By linking laboratory diagnostics with systems biology, multi-omics AI has the potential to transform haematology from a largely descriptive discipline into a predictive and personalised science. However, realising this vision will require robust computational infrastructure, standardised data integration frameworks, and inclusive datasets that reflect global population diversity.

### Conclusion

Artificial intelligence has emerged as a transformative force in haematology laboratory diagnostics, offering measurable improvements in accuracy, reproducibility, and efficiency across morphological assessment, flow cytometry, cytogenetics, genomics, and CDS. These advances signal a shift toward data-driven and personalised haematology practice.

Despite significant progress, widespread clinical adoption remains constrained by challenges related to external validation, model explainability, regulatory uncertainty, and unequal access to digital infrastructure. Ethical oversight and economic considerations must therefore guide AI deployment to ensure that technological innovation aligns with equitable healthcare delivery.

In Africa, AI holds exceptional potential to strengthen diagnostic capacity, expand access to advanced haematology services, and promote regional self-sufficiency in laboratory medicine. Through collaborative networks, federated learning frameworks, and context-sensitive policy development, African laboratories can actively contribute to and benefit from global AI advancement.

Ultimately, the successful integration of AI into haematology will depend on sustained collaboration among clinicians, data scientists, policymakers, and technology developers. By fostering transparent, evidence-based, and ethically grounded AI systems, the field can realise the full potential of AI as a trusted partner in haematology diagnostics, improving patient outcomes on both regional and global scales.
